# Responses of Soil Fungal Communities to Lime Application in Wheat Fields in the Pacific Northwest

**DOI:** 10.3389/fmicb.2021.576763

**Published:** 2021-05-20

**Authors:** Chuntao Yin, Daniel C. Schlatter, Duncan R. Kroese, Timothy C. Paulitz, Christina H. Hagerty

**Affiliations:** ^1^Department of Plant Pathology, Washington State University, Pullman, WA, United States; ^2^Department of Botany and Plant Pathology, Oregon State University, Adams, OR, United States; ^3^Wheat Health, Genetics and Quality Research Unit, United States Department of Agriculture – Agriculture Research Service, Pullman, WA, United States

**Keywords:** microbial community, wheat, soil acidification, fungal community analysis, soil health

## Abstract

Liming is an effective agricultural practice and is broadly used to ameliorate soil acidification in agricultural ecosystems. Our understanding of the impacts of lime application on the soil fungal community is scarce. In this study, we explored the responses of fungal communities to liming at two locations with decreasing soil pH in Oregon in the Pacific Northwest using high-throughput sequencing (Illumina MiSeq). Our results revealed that the location and liming did not significantly affect soil fungal diversity and richness, and the impact of soil depth on fungal diversity varied among locations. In contrast, location and soil depth had a strong effect on the structure and composition of soil fungal communities, whereas the impact of liming was much smaller, and location- and depth-dependent. Interestingly, families Lasiosphaeriaceae, Piskurozymaceae, and Sordariaceae predominated in the surface soil (0–7.5 cm) and were positively correlated with soil OM and aluminum, and negatively correlated with pH. The family Kickxellaceae which predominated in deeper soil (15–22.5 cm), had an opposite response to soil OM. Furthermore, some taxa in Ascomycota, such as Hypocreales, *Peziza* and *Penicillium*, were increased by liming at one of the locations (Moro). In conclusion, these findings suggest that fungal community structure and composition rather than fungal diversity responded to location, soil depth and liming. Compared to liming, location and depth had a stronger effect on the soil fungal community, but some specific fungal taxa shifted with lime application.

## Introduction

Acidic soils are widespread in the world and it is estimated that acidic soils impact over 70% of cultivable and potentially arable land (von Uexküll and Mutert, 1995; Baligar et al., 2001). Soil acidification is a major environmental challenge for crop production in the Inland Pacific Northwest (IPNW) (McFarland and Huggins, 2015; Ghimire et al., 2017), and is a major constraint on plant productivity (Koenig et al., 2013). Continuous use of ammonium-based nitrogen fertilizers and long-term continuous crop growth has caused soil pH to drop below optimum levels for crop production (Schroder et al., 2011; Jones et al., 2019). With soil acidification, pH values below 5.5 increase the solubility and concentration of aluminum (Al^3+^) and manganese (Mn^2+^) in soil which can cause phytotoxicity and reduced crop yields (Kidd and Proctor, 2001; Schroder et al., 2011; Schroeder and Pumphrey, 2013; Lewis et al., 2018). In addition, lower pH also reduces water uptake and soil nutrient availability for plants (Kemmitt et al., 2006). To alleviate the harmful effects of low soil pH on crops, lime can be applied to buffer soil acidification, minimize toxic effects of Al^3+^ and Mn^2+^, and increase soil fertility and plant ecosystem health (Holland et al., 2018; Lewis et al., 2018; Schroeder et al., 2018).

Soil acidity, represented by low pH and high concentration of aluminum and manganese in soil, affects the activity, structure and composition of the soil microbiome, which consequently influences soil function (Lauber et al., 2009; Klaubauf et al., 2010; Narendrula-Kotha and Nkongolo, 2017). Soil pH is considered to be one of the most important soil health indicators (Kemmitt et al., 2006; Rousk et al., 2009), strongly influences microbial communities (Thompson et al., 2017; Jin and Kirk, 2018) and impacts the metabolic activity of the soil microbiome (Leprince and Quiquampoix, 1996; Kotsyurbenko et al., 2004; Ye et al., 2012). Lime application in acidic soil increases soil pH, especially in the soil surface (Lewis et al., 2018; Yin et al., submitted manuscript). Numerous studies have examined the impacts of lime on soil microbes and revealed that lime application changed soil microbial composition and regulated their activities and functions (Narendrula-Kotha and Nkongolo, 2017; Lewis et al., 2018; Schroeder et al., 2018; Pang et al., 2019). Our recent study found that liming has a small effect on soil bacterial communities and the impacts were especially prominent in the surface soil, compared with location and soil depth (Yin et al., submitted manuscript). In addition, liming has been shown to effectively inhibit microbial pathogen populations and reduce disease incidence (Jones et al., 1975; Gatch and duToit, 2017). However, a majority of these studies focused on the changes of bacterial communities after lime amendments in agricultural soil, while the impact of lime on fungal communities is less understood.

Fungi are the second most abundant group of soil microbiota in density and share microhabitats with bacteria. Fungi play key roles in the decomposition of plant residues and organic materials (Rousk et al., 2009; Žifčáková et al., 2016), and are mycorrhizal associates (Smith and Read, 1997; Marcot, 2017), plant pathogens (Maron et al., 2011) and pathogen antagonists (Heydari and Pessarakli, 2010). Compared to bacteria, fungi can live in soil environments across a wider range of pH, temperature and C:N ratios (Strickland and Rousk, 2010; Fra̧c et al., 2015). Despite the broad habitat preferences of fungi, soil acidification may change the composition of the soil fungal community, and consequently, affect the transformation and availability of nutrients for plants (Kemmitt et al., 2006; Al-Sadi and Kazerooni, 2018; Ning et al., 2020). Previous studies on investigating the effects of liming on the fungal community reported conflicting results. For example, Bothe (2015) and Wan et al. (2019) found that liming changed both bacterial and fungal communities. On the contrary, Pennanen et al. (1998) and Pawlett et al. (2009) revealed that liming reduced bacterial phospholipid fatty acids (PLFAs) but did not impact fungal PLFAs. To date, the knowledge of soil fungi response to liming is very limited which makes it difficult to understand the roles of liming on fungal communities in acidic soil.

In this study, we established winter wheat plots in two precipitation zones in Oregon, applied four different rates of lime, and conducted Illumina Miseq to explore the response of the soil fungal community to lime amendments. The objectives of this study were to investigate the impacts of liming, location, and soil depth on the soil fungal community over 2 years. We hypothesized that (1) liming will significantly influence the soil fungal community composition and diversity; and (2) location and soil depth will modulate the impacts of liming on soil fungal communities.

## Materials and Methods

### Study Site and Experimental Design

This study was conducted in two wheat field sites in Oregon within the IPNW, United States which are Columbia Basin Agricultural Research Center (CBARC) in Pendleton (hereafter Pendleton) and the CBARC Sherman Station in Moro (hereafter Moro). Pendleton is in Umatilla County, OR (45.718874, -118.624236) and Moro is in Sherman County, OR (45.483149, -120.725561). The dominant soil type at both locations was a Walla Walla silt loam: coarse-silty, mixed, and mesic Typic Haploxerolls (Web Soil Survey, 2013) and soil pH was below 5.2 in the top 15 cm. The annual precipitation at Pendleton (442 mm) was higher than at Moro (282 mm).

Total field size was 58.5 m wide by 60.9 m long and the experimental plot size within the field was 7.3 m wide by 15.2 m long. Each study site had 16 plots (four treatments × four replicates) which were randomly arranged. Thus, a total of 32 plots were established in two study sites. Winter wheat cv. “Stephens” was seeded with a no-till plot drill 39 and 86 kg/ha urea was added at planting at the Moro and Pendleton locations, respectively. Before planting, four different rates (0, 673, 1,345, and 2,690 kg ha^–1^) of ultrafine liquid calcium carbonate (CaCO_3_, NuCal, Columbia River Carbonates, Vancouver, WA) were applied to surface soil using a custom sprayer outfitted with a Boom Buster (Millen, Georgia) spray nozzle designed to spray a 7.6-m swath in fall, 2016. Within 7 days of the application the plots were subject to light vertical tillage with a Turbo Max (Great Plains Ag. Inc., Salina KS) implement at 0° at both locations. Minimal tillage was conducted at both locations. All plots were managed using an appropriate fertility program including nitrogen and other nutrients based on soil test reports. Weed management and pest control were typical for production of winter wheat in this region.

### Soil Sampling and Chemical Analyses

Soil samples were collected from the two study sites in April 2017 and March 2018, respectively. Soil samples were taken down to a depth of 22.5 cm using a 2.5 cm diameter hand soil probe. The soil core was removed from the probe and separated based on depth: 0–7.5 cm, 7.5–15 cm, and 15–22.5 cm. Each lime treatment consisted of a composite of four soil cores. A total of 48 composite samples per location each year were divided into two portions and stored at −20°C for further analysis.

One portion of the soil samples was sent to a commercial soil lab (Best-Test Analytical Services, Moses Lake, WA) for soil physiochemical characteristics analysis as described by Schroeder et al. (2018). Analyses conducted in this study included organic matter (OM), pH, exchangeable bases (Ca, Mg, and Na), DTPA extractable aluminum (DTPA-Al), KCl extractable aluminum (KCl-Al), Cl, SO_4_.S, B, Zn, Mn, Cu, Fe, N, P, K, and the cation exchange capacity (CEC). Briefly, soil organic matter (OM) was determined using Walkley-Black titration. Exchangeable bases (Ca, Mg, and Na) and Al were extracted with KCl and measured by mass spectrophotometry. The concentrations of Al, SO_4_.S, B, Zn, Fe, Cu, and Mn were extracted with diethylenetriaminepentaacetic acid (DTPA) and measured by mass spectrophotometry. The quantities of K and P were determined using the Olson method, nitrate nitrogen was measured using the chromotrophic acid method, and the cation exchange capacity (CEC) was estimated using the ammonium replacement method.

### DNA Extraction and Illumina Sequencing

Microbial DNA was extracted from 0.25 g of the second portion of soil samples (stored for 2 weeks at −20°C) using a MoBio PowerSoil kit (MoBio/Qiagen, Carlsbad, CA) following the manufacturers’ instruction. Homogenization of soil samples was performed with a FastPrep bead beater (MP Biomedical, Santa Ana, CA) using the “soil” program. The DNA was quantified using a Nanodrop spectrophotometer (Thermo Fisher Scientific, Waltham, MA) and sent to the University of Minnesota Genomics Center (UMGC) for amplification and sequencing. PCR amplification used a dual-indexing approach, as described previously (Gohl et al., 2016). Briefly, the fungal ITS1 region was amplified in the first round of PCR using primers ITS1^∗^_Nextera (5′-TCGTCGGCAGCGTCAGATGTGT ATAAGAGACAGCTTGTCATTTAGAGGAAG^∗^TAA-3′) and ITS2 (5′-GTCTCGTGGGCTCGGAGATGTGTATAAGAGACA GGCTGCGTTCTTCATCGA^∗^TGC-3′). The first PCR consisted of an initial denaturing at 95°C for 5 min, followed by 25 cycles of 98°C for 20 s, 55°C for 15 s, and 72°C for 1 min, with a final extension at 72°C for 5 min. The products from the first PCR were diluted 1:100, and 5 μl was included in a second PCR using forward indexing primer (5′-**AATGATACG GCGACCACCGAGATCTACAC**[i5]TCGTCGGCAGCGTC-3′) and reverse indexing primer (5′-**CAAGCAGAAGACGGCA TACGAGAT**[i7]GTCTCGTGGGCTCGG-3′) (i5 and i7 refer to the index sequence codes used by Illumina. The flow cell adapters are in bold). The second PCR consisted of an initial denaturation at 95°C for 5 min, followed by 10 cycles of 98°C for 20 s, 55°C for 15 s, and 72°C for 1 min, with a final extension at 72°C for 5 min. The products were pooled, size selected, and spiked with 20% PhiX prior to sequencing with an Illumina MiSeq 600 cycle version 3 kit. qPCR was performed to quantify copy numbers of bacterial community from each sample with 16S rRNA primers Meta_V4_515F (5′-TCGTCG GCAGCGTCAGATGTGTATAAGAGACAG**GTGCCAGCMGC CGCGGTAA**-3′) and Meta_V4_806R (5′- GTCTCGTGGG CTCGGAGATGTGTATAAGAGACAG**GGACTACHVGGGTW TCTAAT**-3′) (16S-specific portion in bold) and ITS primers ITS1-F (5′-TCGTCGGCAGCGTCAGATGTGTATAAGAGAC AG**CTTGGTCATTTAGAGGAAG^∗^TAA**-3′) and ITS2 (5′-GT CTCGTGGGCTCGGAGATGTGTATAAGAGACAG**GCTGCG TTCTTCATCGA^∗^TGC**-3′) (ITS-specific portion in bold). qPCR conditions for 16S RNA and ITS consisted of an initial denaturation at 95°C for 5 min, 35 cycles of 98°C for 20 s, 55°C for 15 s and extension at 72°C for 60 s, with the final extension at 72°C for 5 min. The raw sequence data was deposited in OSFHOME^1^.

### Sequence Processing

The sequence processing was conducted using USEARCH (version 11; 42) to denoise sequences and define operational taxonomic units (OTUs). Briefly, primer and barcode sequences were removed along with 50 and 75 base pairs from the 3′ end of forward and reverse reads, respectively. Reads were paired with 15 maximum differences and an 80% identity threshold, followed by the removal of conserved regions at the forward and reverse ends of reads (81 and 68 base pairs, respectively). To generate high-quality reads for denoising, reads were filtered with a maximum expected error rate of 1, singletons were removed, and sequences were denoised using the “unoise3” algorithm. Processed reads were then mapped to OTU representatives to generate an OTU abundance table. Taxonomy was assigned to OTUs using the SINTAX algorithm with an 80% confidence threshold to the SINTAX formatted UNITE database (version 7). OTUs identified as non-fungal were discarded and OTU tables were rarefied to 16,000 sequences per sample for all analyses unless otherwise noted. Material-free extraction controls indicated minimal cross-contamination of fungal sequences in OTU tables prior to rarefaction, thus no removal of contaminant OTUs was necessary.

### Statistical Analyses

The ratio of fungi and bacteria was calculated using copy number of bacterial and fungal community from each sample and compared among treatments by Kruskal-Wallis test. Non-metric multidimensional scaling (NMDS) was performed to visualize fungal community similarity among soil samples based on Bray-Curtis distances using the “metaMDS” function of the vegan package (version 2.4.1) in R. Soil chemical characteristics were fitted to the NMDS ordination with the “envfit” function of vegan. Permutational multivariate analysis of variance (PERMANOVA) was conducted using the “Adonis” function of the vegan package (nperm = 999). Fungal richness and Shannon diversity (H’) were calculated and compared among treatments by Kruskal-Wallis test. The abundance of fungal families (>0.1% of sequences) was compared among treatments by Kruskal-Wallis test. DESeq2 was used to identify OTUs that differed after lime application within soil depth and location using unrarefied OTU tables filtered to remove low abundance taxa (<10 total counts) and those found in fewer than 3 samples. OTUs were considered as differentially abundant if they had a base mean >50, FDR adjusted *p*-values of <0.1, and estimated log2-fold change >1. Spearman correlations were used to evaluate relationships between fungal families and soil chemical characteristics using the “corr.test” function in the Hmisc package in R.

## Results

### Soil Fungal Community

After sequence data processing, 5,460,052 sequences were obtained with a minimum of 16,000 sequences per sample. These sequences were mapped to 4,824 OTUs prior to rarefaction. After rarefaction, the most dominant phylum in soil fungal community was Ascomycota (37.3 ± 0.47%, mean ± SE), followed by Mortierellomycota (13.9 ± 0.49%), and Basidiomycota (13.1 ± 0.49%). Ten other phyla, Chytridiomycota, Calcarisporiellomycota, Kickxellomycota, Rozellomycota, Olpidiomycota, Basidiobolomycota, Glomeromycota, Zoopagomycota, Monoblepharomycota, and Mucoromycota were also present in the test soil but at a lower frequency (less than 2.0%). The four most abundant families comprised over 26% of taxa in all samples, which were Mortierellaceae (13.8 ± 0.49%), Aspergillaceae (5.6 ± 0.27%), Piskurozymaceae (4.4 ± 0.19%), and Herpotrichiellaceae (2.7 ± 0.15%).

**FIGURE 1:**
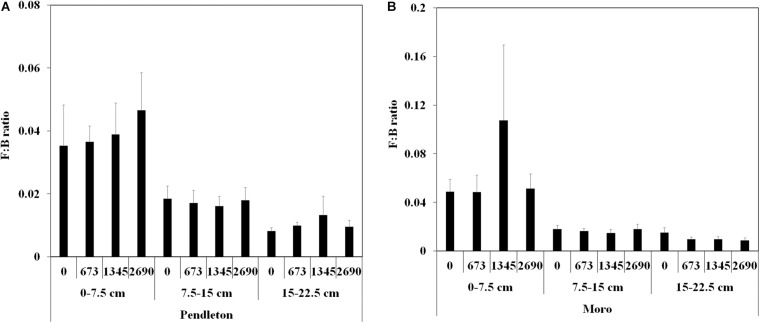
Fungal: bacterial abundance ratios from soil at two wheat locations. **(A)** Pendleton. **(B)** Moro. F:B ratio: fungal: bacterial abundance ratio; Soil depth: 0–7.5 cm, 7.5–15 cm, and 15–22.5 cm; rate of lime application: 0, 673, 1,345, and 2,690 kg ha^–1^; The values are means (*n* = 8) ± SE (*p* ≤ 0.05, Kruskal-Wallis test).

Fungal to bacterial abundance ratios (F:B) were higher in the surface soil (0–7.5 cm) than in the deeper soil (7.5–15 and 15–22.5 cm) at both locations (*p* ≤ 0.05, Kruskal-Wallis test) (Figure 1). But no significant difference of F:B ratio was observed between lime and no lime applications in each soil depth at two locations.

**FIGURE 2:**
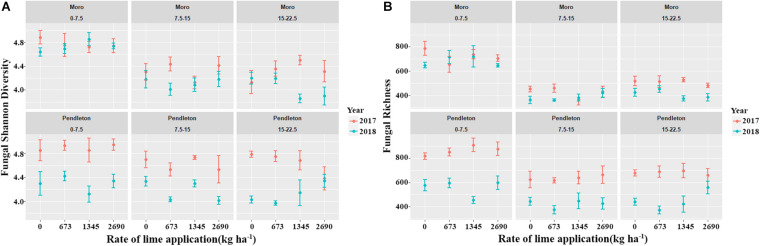
Fungal diversity and richness indices among locations, soil depth, years, and lime applications. **(A)** Fungal Shannon diversity. **(B)** Fungal richness (*p* ≤ 0.05, Kruskal-Wallis test).

Fungal diversity and richness varied between two sampling times (year 2017 and 2018) (*p* ≤ 0.05, Kruskal-Wallis test) (Figure 2). However, lime applications did not significantly impact fungal diversity and richness, compared with the no lime control treatment. In addition, fungal diversity and richness from the surface soil were significantly higher than those of the deeper soils at Moro, and only fungal richness from the surface soil was higher than that of the deeper soil at Pendleton (*p* ≤ 0.05, Kruskal-Wallis test), but no difference of fungal diversity was observed. These results indicate that liming, location, and soil depth had marginal and inconsistent impacts on fungal diversity, while time after liming had greater influence on fungal diversity.

**FIGURE 3:**
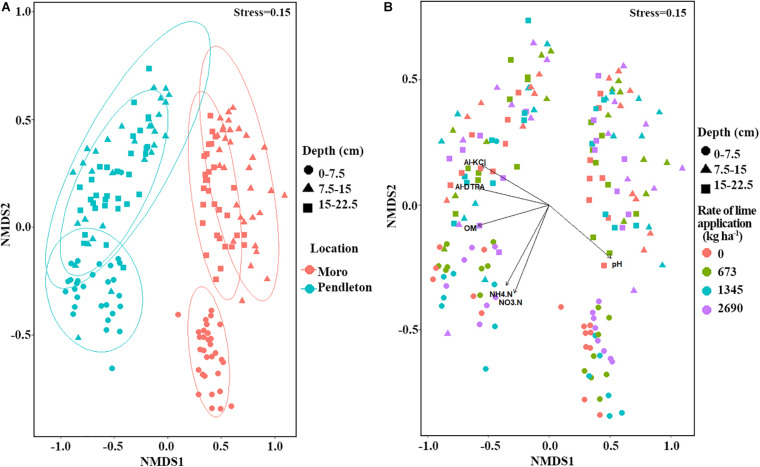
Non-metric multidimensional scaling (NMDS) of fungal community from all samples (Bray-Curtis distances). **(A)** NMDS of fungal community within location and soil depth. **(B)** NMDS of fungal community within soil depth and lime application. Symbols represent soil depth and colors represent location or rate of lime application. Vectors represent significant associations of soil chemical factors along with axes (vegan, *p* ≤ 0.05). Al-DTPA, DTPA extractable aluminum; Al-KCl, KCl extractable aluminum.

**FIGURE 4:**
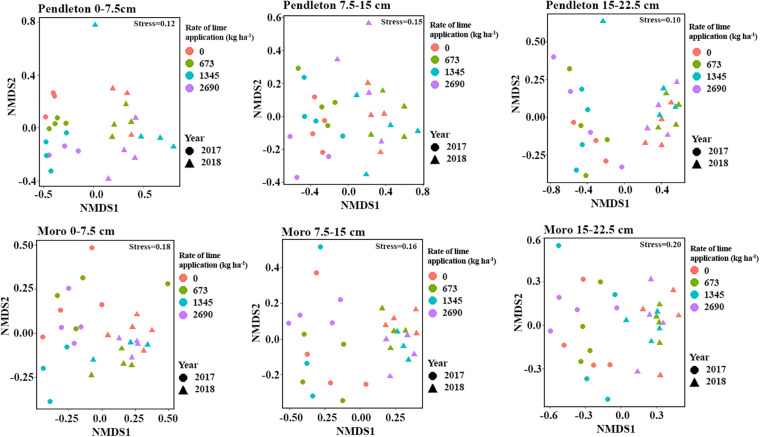
Non-metric multidimensional scaling (NMDS) of fungal community within locations and soil depth (Bray-Curtis distances). Symbols represent sample time after liming (year), and colors represent rate of lime application (kg ha^–1^).

Non-metric multidimensional scaling (NMDS) and PERMANOVA analyses of fungal community composition revealed significant location (PERMANOVA *r*^2^ = 0.19, *p* = 0.001) and soil depth (PERMANOVA *r*^2^ = 0.15, *p* = 0.001) effects (Figure 3A). A small but significant effect of liming (PERMANOVA *r*^2^ = 0.02, *p* = 0.015) on fungal community composition was observed (Figure 3B). Additionally, fungal community dissimilarities were significantly correlated with soil chemical parameters, including organic matter (OM), DTPA-Al, KCl-Al, pH, and nitrogen (NH_4_.N and NO_3_.N) (Figure 3B). Further, fungal communities from different locations and soil depths were analyzed independently using NMDS. The results showed that time after lime treatment (year) influenced fungal composition (PERMANOVA, *p* = 0.001). The impacts of liming on fungal community composition were only observed in the surface soil at Moro (PERMANOVA *r*^2^ = 0.12, *p* = 0.008), but not in the surface soil at Pendleton or in deeper soils at either location (*p* > 0.27) (Figure 4).

### Effect of Location and Soil Depth on Fungal Taxa

Differences in the relative abundance of fungal families by location and soil depth were observed in soil samples without liming (*p* ≤ 0.05, Kruskal-Wallis test). Eighteen and 14 fungal families were significantly affected by location and soil depth, respectively, where some families were more abundant in the surface soil (0–7.5 cm) (e.g., Coniochaetaceae, Dothioraceae, Lasiosphaeriaceae, Piskurozymaceae, and Sordariaceae), some in deeper soil (15–22.5 cm) (e.g., Calcarisporiellaceae, Geminibasidiaceae, Kickxellaceae, and Mortierellaceae), and some families (Aspergillaceae, Hypocreaceae, and Myxotrichaceae) in the middle layer (7.5–15 cm) (Table 1). Moreover, nine families varied significantly with both location and soil depth.

**TABLE 1:** The relative abundance (±standard error) of fungal families significantly impacted by location and soil depth without liming.

Family (relative abundance%)	Pendleton (cm)			Moro (cm)			*p*-value^a^ (location)	*p*-value^a^ (soil depth)
		
	0–7.5	7.5–15	15–22.5	0–7.5	7.5–15	15–22.5		
Aspergillaceae	1.960.20	4.780.52	4.080.88	5.740.84	11.111.11	5.810.93	9.11E-05	1.53E-02
Calcarisporiellaceae	0.020.01	1.710.31	3.491.29	0.000.00	0.080.05	0.230.12	2.39E-04	2.24E-04
Cantharellales_fam_Incertae_sedis	0.010.01	0.000.00	0.050.03	0.150.07	6.573.71	3.723.29	4.87E-05	>0.05
Ceratobasidiaceae	0.300.15	0.340.10	0.720.28	0.080.04	0.160.08	0.120.10	7.21E-04	>0.05
Chaetomiaceae	0.190.07	0.230.12	0.230.07	0.790.16	0.720.17	0.500.28	1.39E-03	>0.05
Clavicipitaceae	0.140.09	0.590.19	0.570.20	0.050.01	0.620.15	0.590.27	>0.05	9.36E-06
Coniochaetaceae	2.080.61	0.280.05	0.440.15	1.920.57	0.420.13	0.340.04	>0.05	4.43E-06
Cystofilobasidiaceae	0.410.10	0.250.17	0.250.13	0.000.00	0.010.00	0.000.00	3.53E-08	>0.05
Dothioraceae	0.020.01	0.000.00	0.010.01	0.810.16	0.240.08	0.490.06	1.60E-07	3.97E-02
Geminibasidiaceae	0.020.01	0.210.04	0.650.16	0.080.02	1.120.28	2.200.57	6.11E-03	1.20E-06
Herpotrichiellaceae	1.180.15	1.390.19	1.980.50	4.900.73	3.120.65	4.520.77	1.77E-06	>0.05
Hypocreaceae	0.620.37	1.800.38	0.760.15	0.130.03	0.590.18	0.390.18	6.16E-04	2.33E-03
Kickxellaceae	0.010.01	0.140.07	1.941.14	0.050.03	0.460.19	0.570.24	>0.05	7.07E-04
Lasiosphaeriaceae	2.510.46	0.740.32	0.550.25	0.740.25	0.340.14	0.180.11	1.80E-02	1.67E-03
Mortierellaceae	7.091.29	15.851.34	16.451.84	8.781.15	14.042.27	16.372.35	>0.05	6.49E-05
Myxotrichaceae	0.020.01	0.150.06	0.050.02	0.080.03	0.200.08	0.090.05	>0.05	7.56E-03
Orbiliaceae	0.010.00	0.010.00	0.080.03	0.200.02	0.050.02	0.230.09	6.77E-05	5.96E-03
Piskurozymaceae	5.630.95	7.891.21	4.520.61	3.690.27	3.550.52	1.640.31	9.10E-05	7.18E-03
Sordariaceae	3.861.10	0.820.22	0.760.42	0.040.02	0.050.03	0.040.03	3.65E-07	>0.05
Teratosphaeriaceae	0.000.00	0.000.00	0.000.00	0.570.12	0.360.24	0.480.14	7.34E-09	>0.05
Xylariaceae	0.010.00	0.000.00	0.010.01	0.460.25	0.220.15	0.020.01	8.05E-05	>0.05
Chaetosphaeriaceae	0.590.18	0.780.07	0.760.08	0.080.02	0.660.28	0.960.61	1.33E-03	3.04E-02
Helotiaceae	0.030.01	0.100.05	0.550.47	0.130.05	0.230.08	0.290.09	1.15E-02	>0.05

**^*a*^Kruskal-Wallis test.**

### Effect of Lime Applications on Fungal Taxa

**FIGURE 5:**
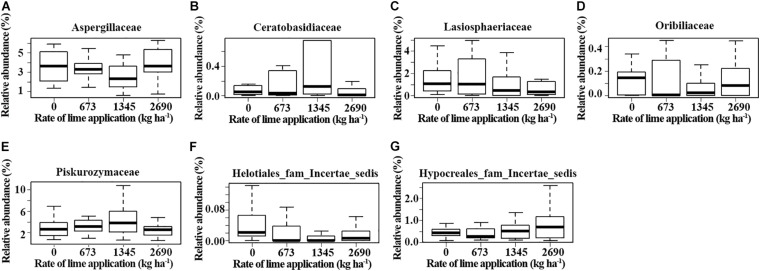
Fungal families that were impacted by lime application at different soil depths. **(A–D)** 0-7.5 cm soil, **(E)** 7-15 cm soil, and **(F–G)** 15-22.5 cm soil. Box: interquartile range. Mean: the heavy line in the box (*p* ≤ 0.05, Kruskal–Wallis test).

**FIGURE 6:**
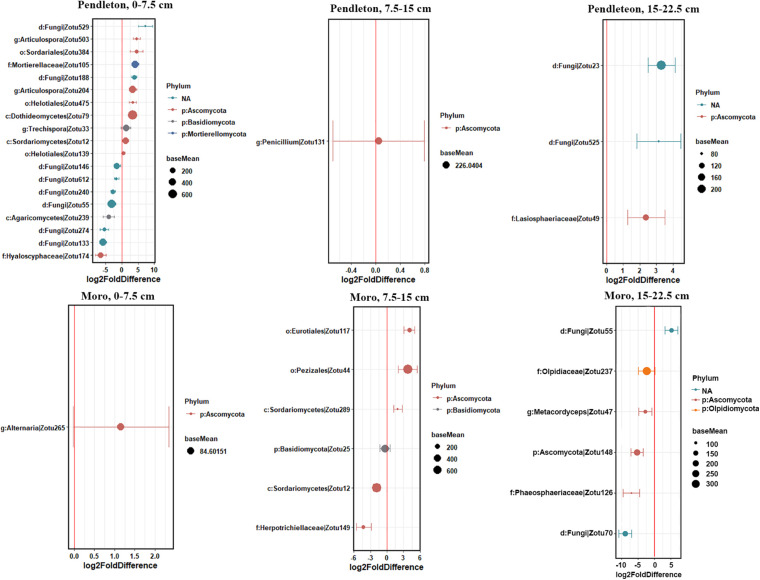
Fungal OTUs that were differentially abundant among lime application within soil depth at each location. Soil depth: 0–7.5 cm, 7.5–15 cm, and 15–22.5 cm. Values on the X-axis represent the log2-fold difference in sequence abundance among lime amendments as estimated by DESeq2. Points to the right of red vertical line are more abundant in lime amendments, whereas those to the left of red vertical line are less abundant in lime amendments. Dots indicate OTUs, where the size of the dot is scaled by its mean abundance among all samples (baseMean > 50) and its color represents the phylum to which that OTU belongs. The nearest taxonomy assignment is presented at left. Only OTUs with a mean abundance >10 and normalized counts >5 and present in at least 3 samples are presented.

Some fungal families varied in relative abundance with lime application in different soil depths at each location (*p* ≤ 0.05, Kruskal-Wallis test) (Figure 5). These families included Chaetosphaeriaceae, Helotiaceae and Lasiophaeriaceae at Pendleton, and Aspergillaceae, Lasiophaeriaceae, Pezizaceae and Thelebolaceae at Moro in the surface soil. Notably, the abundance of Helotiaceae and Pezizaceae increased with rate of lime application, while Lasiophaeriaceae showed an opposite trend. The abundance of Dothioraceae and Helontialea_fam_Incertae_sedis at Pendleton and Entolomataceae and Helontialea_fam_Incertae_sedis at Moro varied with lime application in middle layer (7.5–15 cm). Families Herpotrichiellaceae and Orbiliaceae varied with lime application in deeper soil (15–22.5 cm) at Pendleton. No family in deeper soil at Moro was affected by liming. Furthermore, DESeq2 analysis identified 53 (0–7.5 cm soil), 5 (7.5–15 cm soil), and 6 (15–22.5 cm soil) OTUs at Pendleton, and 12 (0–7.5 cm soil), 22 (7.5–15 cm soil), and 6 (15–22.5 cm soil) OTUs at Moro that differed in abundance after liming application (base mean > 50) (Figure 6 and Supplementary Figure 1). A total of 32 OTUs increased and 32 OTUs decreased by liming at Pendleton, and 20 OTUs increased and 20 OTUs decreased by liming at Moro, respectively. However, most OTUs impacted by liming were soil depth- or location-dependent, where OTU 148 had a consistent negatively response to liming in both 7.5–15 cm and 15–22.5 cm soil at Moro, and OTU 32 had a consistent positive response to liming in surface soil at Pendleton and middle layer soil at Moro, while OTU 49 (Lasiosphaeriaceae) exhibited an opposite response to liming in 7.5–15 cm and 15–22.5 cm soil at Pendleton (Figure 6 and Supplementary Figure 1). Interestingly, several fungal taxa among these OTUs known for their roles as pathogens, such as Herpotrichiellaceae, *Alternaria*, and Sordariomycetes, were found. On the other hand, a few taxa were reported to have members with biocontrol activities, such as Hypocreales and *Mortierella*. Moreover, a few genera, including *Trichoderma* and OTU 127 (order Hypocreales), *Peziza* (order Pezizales), and *Penicillium* (order Eurotiales), in Ascomycota were increased by liming at Moro location. Taken together, these results indicate that the impacts of liming on soil fungal taxa were inconsistent and location- and soil depth- dependent.

### Relationships of Fungal Communities With Soil Chemical Properties

**FIGURE 7:**
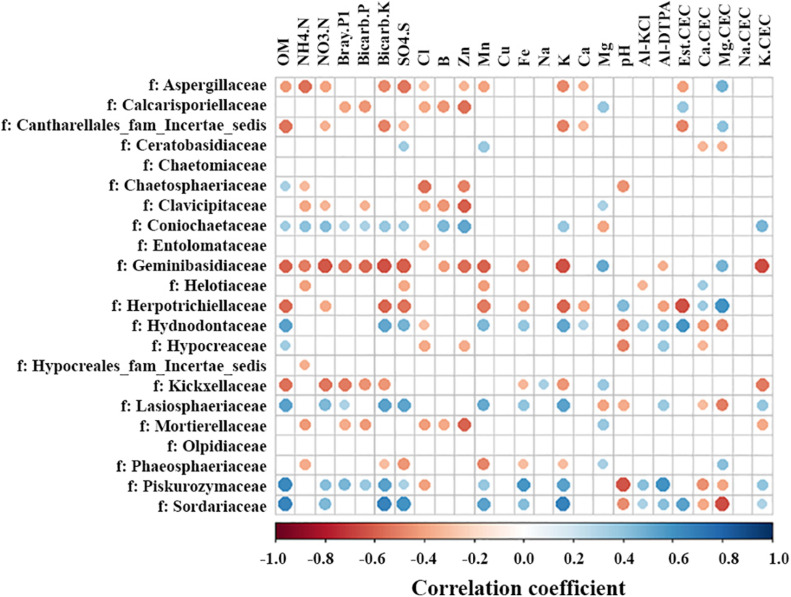
Heatmap of significant spearman correlations between fungal family abundance and soil chemical characteristics. OM, organic matter; Al-KCl, KCl extractable aluminum; Al-DTPA, DTPA extractable aluminum; CEC, cation exchange capacity and the element listed before CEC (i.e., Ca.CEC) represents the proportion of the CEC comprised of that specific cation. Significance was examined using R statistical software (*p* ≤ 0.05 and absolute *r*-value cut off 0.3).

Lime application changed soil chemical properties, including increasing pH and the levels of Ca^2+^ and calcium cation exchange capacity (Ca. CEC) (Yin et al., submitted manuscript, Supplementary Figures 2, 3). Additionally, a strong negative correlation was observed between the organic matter (OM) and soil depth (Supplementary Figure 3). The impacts of soil chemical properties on fungal families were analyzed. Most interestingly, the response of some fungal families to soil OM and pH was opposite (Figure 7). For example, fungal families were positively correlated with soil OM and negatively with pH, including Chaetosphaeriaceae, Hydnodontaceae, Hypocreaceae, Lasiosphaeriaceae, Piskurozymaceae, and Sordariaceae. Whereas Herpotrichiellaceae was negatively correlated with OM and positively correlated with pH. Compared with pH, six of seven families had opposite correlations with Al-KCl or Al-DTPA or both (Figure 7). Further, the correlations between soil chemical properties and fungal families from different soil depths were analyzed independently (Supplementary Figure 4). Similar trends were found although the opposite response of a few fungal families to OM and pH was observed only in the surface (e.g., Lasiosphaeriaceae) or deeper soil (e.g., Calcarisporiellaceae and Hydnodontaceae). In addition, other soil characteristics, such as nitrogen (NH_4_. N and NO_3_.N), P, K, and cation exchange capacity, had positive or negative correlations with fungal families (Figure 7). These results suggest that soil characteristics are important drivers of the fungal community.

## Discussion

Few studies have addressed the impact of lime application in agricultural soil on the soil fungal community. We investigated the changes in the soil fungal community after liming at two locations with decreasing soil pH in the IPNW using high-throughput sequencing. Contrary to bacterial communities (Yin et al., submitted manuscript), location and liming did not significantly influence fungal diversity and richness in soil, while the impacts of soil depth on fungal diversity were location-dependent. These results were consistent with the work by Narendrula-Kotha and Nkongolo (2017) who showed that soil liming had limited effects on fungal diversity. They were also in agreement with work by Al-Sadi and Kazerooni (2018) who reported that no significant differences of Chao richness and Shannon diversity existed in root samples taken from limed soils. However, this is not always the case for fungal diversity. Lin et al. (2018) showed that lime or pig manure altered fungal community diversity due to soil pH increase in Ultisols. Generally, fungi can live in a wider range of pH than bacteria (Strickland and Rousk, 2010). In our study sites, liming ameliorated soil acidification in the surface soil (0–7.5 cm) and soil pH slightly increased with soil depth in both locations (Yin et al., submitted manuscript, Supplementary Figure 2). Increasing pH by liming or soil depth may benefit some fungi which are favored by relatively higher pH conditions (Rousk et al., 2010). On the other hand, increased pH may suppress the growth of other fungi which are well-adapted to acidic soil (Dix and Webster, 1995). Consequently, fungal diversity appeared to be unaffected by location and liming.

Contrary to fungal diversity, fungal community composition was significantly influenced by location and soil depth. Notably, stratification of fungal families was observed, where families such as, Coniochaetaceae, Dothioraceae, Lasiosphaeriaceae, Piskurozymaceae, and Sordariaceae tended to predominate in surface soil, whereas families Calcarisporiellaceae, Clavicipitaceae, Geminibasidiaceae, Kickxellaceae, and Mortierellaceae predominated in deeper soil. Moreover, the surface soil enriched families, Lasiosphaeriaceae, Piskurozymaceae, and Sordariaceae, were positively correlated with soil OM and aluminum, and negatively with pH, whereas deeper soil enriched family Kickxellaceae showed an opposite correlation with soil OM. OM was higher in the surface soil and decreased with soil depth in our study sites. Plant residue and litter layer may result in increased OM in the surface soil, while plant residue is expected to be reduced with depth, which is likely to be reflected by less OM in deeper soil. Similar to OM, soluble aluminum was negatively correlated with soil depth, but pH showed an opposite trend with lower pH in the surface and higher pH in deeper soil. These results suggested that variation in fungal families across soil depths may track stratification in OM, pH, and aluminum in soil. Further, earlier studies also supported these findings. For example, fungal community composition was found to be distinct at different soil depths (Ko et al., 2017). Li et al. (2017) revealed that the relative abundances of bacteria and fungi significantly decreased at 0–40 cm depths. Similarly, fungal community varied with the soil depths (Prober et al., 2015; Schlatter et al., 2018; Guo et al., 2019). In addition, Schlatter et al. (2018) found that family Lasiosphaeriaceae was more abundant at soil surface, while Clavicipitaceae were in deeper soil (10–25 cm). Taken together, stratification of OM, aluminum, and pH across soil depths may generate distinct fungal communities.

Similar to the small impacts of liming on bacterial community observed in our companion work (Yin et al., submitted manuscript), we found a subtle effect of liming on fungal community composition. However, liming influenced the abundance of 11 fungal families in different soil depths at two locations and seven families had strong correlations with soil pH (Figures 5, 7). Most notably, the abundance of family Lasiosphaeriaceae (phylum Ascomycota) decreased with greater lime concentration in the surface soil and had negative correlations with soil pH. Liming significantly increased soil pH in the surface soil at two locations, suggesting that liming decreased the abundance of Lasiosphaeriaceae through increasing soil pH. Liming also decreased aluminum concentration in the surface soil, and significantly increased soil calcium concentration (Yin et al., submitted manuscript) which may reduce the solubility of OM (Balaria et al., 2014). The abundance of Lasiosphaeriaceae was positively correlated with soil OM and aluminum (Figure 7), which could explain the abundance of Lasiosphaeriaceae which decreased with greater lime concentration in the surface soil. In addition, some Ascomycetes are important sources of extracellular enzymes (Moorhead et al., 2012) and liming may decrease the activities of some extracellular enzymes (Cenini et al., 2016; Sridhar et al., 2018), which may be the other reason for less abundant Lasiosphaeriaceae in the surface soil. Similar trends were also observed in the abundance of family Chaetospheriaceae in the surface soil at Pendleton. In contrast, the abundance of Helotiaceae at Pendleton and Pezizaceae at Moro in the surface increased with the rate of lime application. Interestingly, Helotiaceae was positively correlated with soil Ca, but negatively correlated with soil Al, indicating the increase of Helotiaceae with the rate of lime application through increasing soil Ca and decreasing Al after liming. In addition, Helotiaceae was negatively correlated with soil NH_4_. Helotiaceae is a small group of fungicolous, lichenicolous, and discomycetes members (Zhuang, 2000) and was reported to be positively correlated with soil carbon accumulation (Zhang et al., 2017). Nagati et al. (2019) found that higher abundance of Helotiaceae was associated with the lower N nutrition of balsam fir near ericaceous shrubs. Moreover, previous studies reported that soil C:N ratio significantly increased with liming and the effect of liming on soil fungal community was closely tied to the way liming affected the C:N ratio (Melvin et al., 2013; Suz et al., 2014). It is suggested that the abundance of Helotiaceae may be related to soil nutrient is C:N ratio. Tedersoo et al. (2006) revealed that some pezizalean species were distributed in the forests with a high pH, which was consistent with our findings. In addition, it was unexpected that the abundance of ascomycete family Nectriaceae was very low (<0.1%) in our soil. Nectriaceae includes numerous important plant pathogens, such as *Fusarium* spp. soilborne pathogens which are usually very common and associated with plant debris and roots in agricultural soil (Silvestro et al., 2013; Karim et al., 2016). However, the impacts of liming on fungal community were observed in short term (2 years) after lime applications. A few previous studies reported that impact zones with increased pH and the changes of soil chemistry reached to depths of 50, 55, and 60 cm in over 10 years after liming (Tang et al., 2003; Caires et al., 2008; Santos et al., 2018). The microbial properties under longer timescale liming need to be further addressed. In summary, location and soil depth significantly influenced fungal community composition, while lime had a smaller impact. A small number of fungal families varied in relative abundance after liming and liming influenced some fungal family abundance through increasing soil pH.

The ratio of fungal and bacterial abundance (F:B) was considered to have important ecological significance in soil ecology and affected by environmental changes (Strickland and Rousk, 2010; Wang et al., 2019). We found that the ratios of F:B were higher on the surface soil than that of deeper soil at both locations. It is probable that fungi are less affected by soil pH than bacteria (Rousk et al., 2010), thus dominate in surface soil resulting in a higher ratio of F:B than the deeper soil. Another possibility is that there is more plant residue in the surface soil than the deeper soil. Although both bacteria and fungi are decomposers in agro-ecosytems, the roles they play are different. Compared to bacteria, soil fungi mainly decompose recalcitrant organic materials, such as lignin, cellulose, and hemicellulose, whereas bacteria degrade labile fractions (Strickland and Rousk, 2010; Chen et al., 2014). Thus, the surface soil may enrich more fungi than the deeper soil for plant residue decomposition. Finally, the oxygen concentration in the deeper soil is much lower than those in the surface soil which may also change the microbial distribution.

Findings from this study revealed that the most dominant fungal phylum was Ascomycota, accounting for >35% of fungi in examined soil. Ascomycota is the dominant phylum of fungi in agricultural ecosystems and its high presence in our soil is not unexpected. Ascomycota play significant roles in nutrients and carbon cycling and induce the uptake of mineral nutrients for plants (Semenova et al., 2015; Francioli et al., 2016; Yu et al., 2018). The second and third most dominant phyla were Mortierellomycota and Basidiomycota, accounting for similar abundance. *Mortierella* is the largest genus in Mortierellomycota and known as a ubiquitous saprobe (Buée et al., 2009). Basidiomycota, rather than Ascomycota, was dominant in terrestrial ecosystems such as forests but was much lower in agricultural soils (Francioli et al., 2016) which supported our results. Most interestingly, a few genera, including *Trichoderma* and OTU 127 (order Hypocreales), *Peziza* (order Pezizales) and *Penicillium* (order Eurotiales), in Ascomycota were increased by liming at the Moro location. These results were consistent with the works by Narendrula-Kotha and Nkongolo (2017) and Al-Sadi and Kazerooni (2018) who found that *Penicillium* was more abundant in limed soil than a non-limed site. They were partially in accord with (Ye et al., 2020) who revealed that liming significantly enhanced the relative abundance of the order Hypocreales but had no effect on the relative abundance of Pezizales. Similarly, Pezizales are abundant in forest soil and increased with liming (Kjøller and Clemmensen, 2009; Tedersoo et al., 2014; Ge et al., 2017). Earlier studies showed that some fungi, such as Hypocreales and *Peziza*, grow well in neutral to slightly alkaline conditions (Yamanaka, 2003; Rousk et al., 2010). Thus, one possible explanation is that increased pH by liming in our study sites promoted the growth of Hypocreales and *Peziza* and increased their abundance. Some genera of Hypocreales were considered as sources of biocontrol fungi for plant diseases (Bastakoti et al., 2017; Kepler et al., 2017) and *Penicillium* was reported to interfere with the growth of some fungal pathogens (Yuan et al., 2017). Similarly, genus *Mortierella* from Mortierellomycota was slightly increased by liming at Moro. *Mortierella* was reported to be beneficial for plant growth and soil health by releasing P or degrading relative abundances of the toxic compounds and producing antibiotics to inhibit phytopathogens (Tagawa et al., 2010; Osorio and Habte, 2015; Li et al., 2018). A recent study found that the enrichment of *Mortierella* species increased crop yield (Ning et al., 2020). Therefore, the increased abundance of these taxa in limed soil may contribute to pathogen suppression, promote crop growth, thus indirectly increase crop yields. Further work is needed to verify function of *Mortierella* in this system. This phenomenon was only observed at Moro, indicating a location dependency.

## Conclusion

Overall, our findings revealed minor impacts of liming on soil fungal community composition, similar to small effects on bacterial community (Yin et al., submitted manuscript). Location and soil depth had a strong effect on soil fungal community composition. However, the location and liming did not significantly influence soil fungal diversity and richness. Some fungal taxa, including species associated with plant benefits, varied after lime application which may improve plant growth and increase crop yields.

## Data Availability Statement

The names of the repository/repositories and accession number(s) can be found below: https://www.ncbi.nlm.nih.gov/sra/?term=PRJNA678975.

## Author Contributions

CY: analysis, writing, and bioinformatics. DS: bioinformatics. DK: plot and data management. TP: project conception, direction, and personnel management. CH: project conception, direction, funding, personnel management, and writing. All authors contributed to the article and approved the submitted version.

## Conflict of Interest

The authors declare that the research was conducted in the absence of any commercial or financial relationships that could be construed as a potential conflict of interest.

## Footnotes

1 https://osf.io/twux3/?view_only=99b35f5e106542f3b08c7a1c2616f6e0

## Supplementary Material

Supplementary Figure 1 Venn diagram of OTUs influenced by liming treatments among soil depths. **(A)** Pendleton, **(B)** Moro.

Supplementary Figure 2 Soil pH changes after lime amendments at two wheat locations. Soil depth: 0–7.5 cm, 7.5–15 cm, and 15–22.5 cm. The values are means (*n* = 32) ± SE. Different letters represent significant differences (*p* ≤ 0.05, Tukey test).

Supplementary Figure 3 Heatmap of significant Spearman correlations between soil chemical characteristics and soil depth and location. OM, organic matter; Al-DTPA, DTPA extractable aluminum; Al-KCl, KCl extractable aluminum; CEC, cation exchange capacity and the element listed before CEC (i.e., Ca.CEC) represents the proportion of the CEC comprised of that specific cation. Significance was examined using R statistical software (*p* ≤ 0.05 and absolute *r* value cut off 0.3).

Supplementary Figure 4 Heatmap of significant Spearman correlations between fungal family abundance and soil chemical characteristics at different soil depths. OM, organic matter; Al-KCl, KCl extractable aluminum; Al-DTPA, DTPA extractable aluminum; CEC, cation exchange capacity and the element listed before CEC (i.e., Ca.CEC) represents the proportion of the CEC comprised of that specific cation. Significance was determined using R statistical software (*p* ≤0.05 and absolute *r-*value cut off 0.3).
