# Supplementary figures and images for: Responses of Soil Fungal Communities to Lime Application in Wheat Fields in the Pacific Northwest
Source: Front Microbiol. 2021 May 20;12:576763. doi: 10.3389/fmicb.2021.576763 (PMC8174452; doi:10.3389/fmicb.2021.576763)

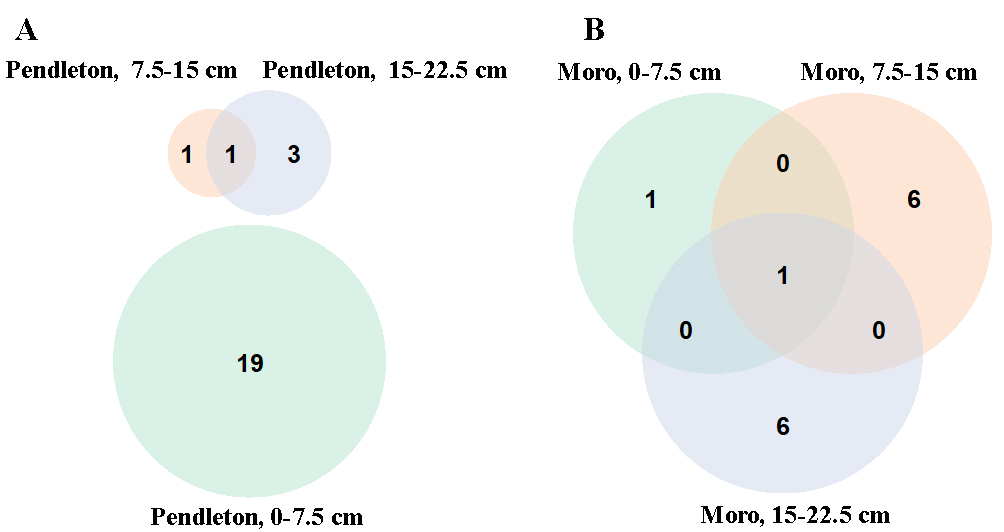

Supplement: Supplementary Figure 1 — Venn diagram of OTUs influenced by liming treatments among soil depths. (A) Pendleton, (B) Moro. [file Image_1.jpeg]

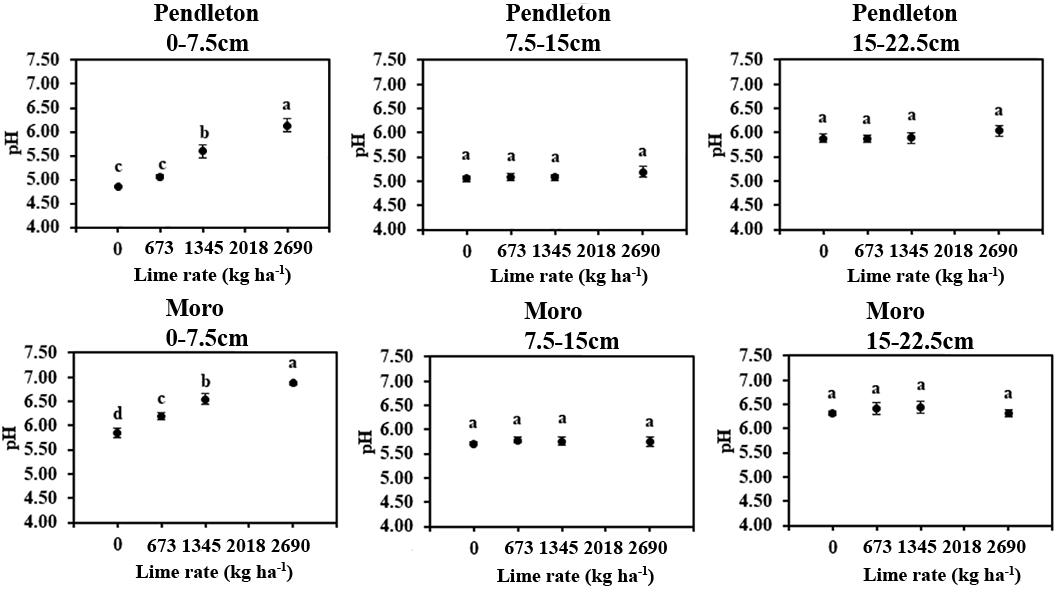

Supplement: Supplementary Figure 2 — Soil pH changes after lime amendments at two wheat locations. Soil depth: 0–7.5 cm, 7.5–15 cm, and 15–22.5 cm. The values are means (n = 32) ± SE. Different letters represent significant differences (p ≤ 0.05, Tukey test). [file Image_2.jpeg]

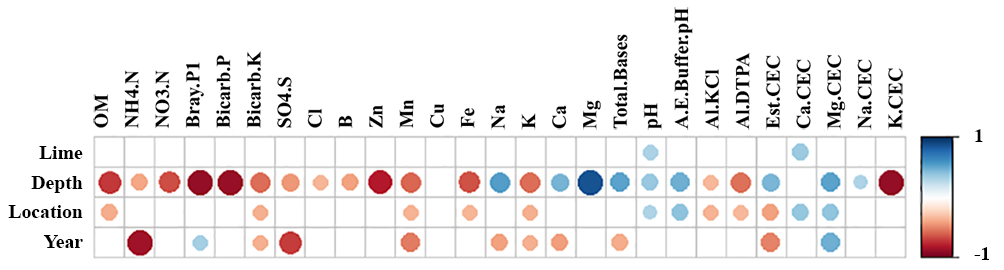

Supplement: Supplementary Figure 3 — Heatmap of significant Spearman correlations between soil chemical characteristics and soil depth and location. OM, organic matter; Al-DTPA, DTPA extractable aluminum; Al-KCl, KCl extractable aluminum; CEC, cation exchange capacity and the element listed before CEC (i.e., Ca.CEC) represents the proportion of the CEC comprised of that specific cation. Significance was examined using R statistical software (p ≤ 0.05 and absolute r value cut off 0.3). [file Image_3.jpeg]

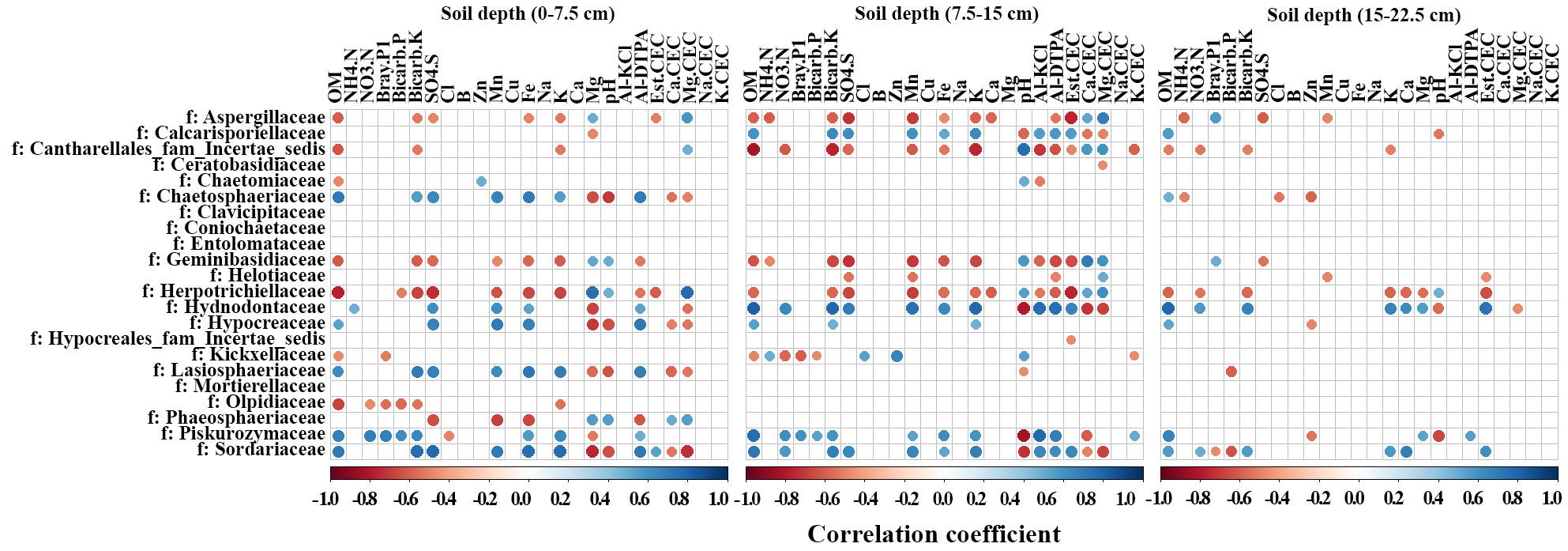

Supplement: Supplementary Figure 4 — Heatmap of significant Spearman correlations between fungal family abundance and soil chemical characteristics at different soil depths. OM, organic matter; Al-KCl, KCl extractable aluminum; Al-DTPA, DTPA extractable aluminum; CEC, cation exchange capacity and the element listed before CEC (i.e., Ca.CEC) represents the proportion of the CEC comprised of that specific cation. Significance was determined using R statistical software (p ≤0.05 and absolute r-value cut off 0.3). [file Image_4.jpeg]
